# Safety and feasibility of paired vagus nerve stimulation with rehabilitation for improving upper extremity function in people with cervical spinal cord injury: a pilot randomized controlled trial

**DOI:** 10.3389/fresc.2026.1805955

**Published:** 2026-06-11

**Authors:** Radha Korupolu, Nuray Yozbatiran, Alyssa Miller, Meredith Shields, Nitin Tandon

**Affiliations:** 1 Department of Physical Medicine and Rehabilitation, McGovern Medical School, The University of Texas Health Science Center at Houston, Houston, TX, United States; 2 TIRR Memorial Hermann Hospital, Houston, TX, United States; 3 Neuromodulation and Neural Interfaces Laboratory, UTHealth NeuroRecovery Research Center at TIRR Memorial Hermann, Houston, TX, United States; 4 Vivian L. Smith Department of Neurosurgery, McGovern Medical School, UTHealth Houston, Houston, TX, United States

**Keywords:** neuromodulation, neurorehabilitation, spinal cord injury, upper extremity function, vagus nerve stimulation

## Abstract

**Background:**

Recovery of upper extremity function remains a major unmet need for individuals with chronic cervical spinal cord injury (SCI). Pairing vagus nerve stimulation (VNS) with task-specific rehabilitation has been shown to enhance neuroplasticity and motor recovery in stroke, but evidence in SCI is limited.

**Objective:**

To evaluate the safety and feasibility of pairing VNS with upper extremity rehabilitation in individuals with chronic cervical SCI, and to explore preliminary effects on upper extremity function.

**Methods:**

This single-site, triple-blind, randomized, sham-controlled pilot trial enrolled adults with chronic traumatic incomplete cervical SCI. All participants underwent implantation of a VNS device and were randomized 1:1 to receive active VNS paired with rehabilitation or sham VNS paired with rehabilitation. Participants completed 18 in-clinic rehabilitation sessions over 6–8 weeks, followed by a 90-day home-based exercise program. Primary outcomes were safety and feasibility; exploratory outcomes included standardized upper extremity motor measures and patient-reported experiences. The randomized design was intended to assess feasibility of trial procedures rather than estimate treatment effects.

**Results:**

Six participants were implanted and completed study procedures through 90-day follow-up. No serious adverse events, unanticipated adverse device effects, or unresolved surgery-related complications were observed. In-clinic therapy adherence was high, supporting the feasibility of the intervention. At the immediate post-intervention, all participants receiving active VNS demonstrated mild-to-moderate improvements in upper extremity motor function, whereas no improvements were observed in the sham group. At 90 days, two active VNS participants sustained these gains. Participants receiving active VNS reported subjective improvements in coordination, movement quality, and daily task performance.

**Conclusion:**

This pilot randomized trial demonstrates that pairing invasive VNS with upper extremity rehabilitation in individuals with chronic cervical spinal cord injury is safe, feasible, and well-tolerated. While clinical improvements were exploratory, positive participant-reported experiences and observed patterns of improvement support further investigation in larger trials designed to rigorously assess efficacy, durability, and optimal dosing parameters.

**Clinical Trial Registration:**

ClinicalTrials.gov, identifier NCT05601661.

## Introduction

The annual incidence of traumatic spinal cord injury (SCI) worldwide was estimated as 768,473 new cases with a 95% confidence interval (CI) of 597,000–939,732 new cases (1); with cervical lesions resulting in complete or incomplete cervical SCI accounting for more than 50% of new cases (2). Cervical SCI causes paralysis of all four extremities (tetraplegia), leading to profound disability due to loss of functional abilities required to live independently, including transfers and basic activities of daily living such as feeding, bathing, grooming, dressing, toileting, bladder, and bowel management (3, 4). Among individuals with tetraplegia, regaining arm/hand function is one of the top priorities (5), as it is crucial for improving their independence and quality of life. A variety of approaches are used to improve upper extremity function after SCI, including exercise, biofeedback, robotic therapy, functional electrical stimulation, task-specific movement therapy, and reconstructive surgeries (6–11). However, recovery is challenging, and people with SCI frequently have residual motor disabilities even after completing conventional rehabilitation therapies.

Regeneration of the damaged spinal cord is limited (12). Yet, current evidence clearly shows that most of the neurologic recovery after SCI is largely mediated by synaptic plasticity within spared neural pathways above and below the level of injury (12–14). Neuroplasticity is the capacity of spared neural cells and pathways to adapt in response to intrinsic and extrinsic factors, and is a critical mechanism for functional recovery following neurological injury (15–17). To maximize recovery beyond that achieved with conventional therapy alone, various invasive and non-invasive neuromodulation interventions have been investigated in combination with rehabilitation. One such emerging approach is vagus nerve stimulation (18).

VNS paired with rehabilitation is a promising intervention for promoting neuroplasticity. The vagus nerve is a cranial nerve that provides parasympathetic and branchial motor efferent activation to several target organs; however, a large portion of the vagus nerve consists of afferent connections to several nuclei in the brain stem. VNS fosters a neurochemical environment that facilitates the release of neuromodulators such as acetylcholine, norepinephrine, serotonin, and brain-derived neurotrophic factors, which promote cortical plasticity (19–24). The repeated pairing of brief bursts of VNS with sensory or motor events produces greater expansion of cortical representations than to interventions without VNS pairing (19, 25). The effects of VNS paired with rehabilitation have been extensively studied in animal models of stroke, traumatic brain injury, and SCI (26–34). These studies consistently demonstrated enhanced cortical plasticity and improved upper extremity recovery with paired VNS rehabilitation, compared to identical rehabilitation without VNS. Pre-clinical animal studies further suggest that dosing and precise timing, specifically rapid bursts of VNS delivered immediately following a motor event at 0.8 mA and 30 Hz, are critical to maximize cortical plasticity and motor recovery (27, 30, 31, 34).

Subsequently, the safety, feasibility, and efficacy of these VNS parameters paired with rehabilitation were proven in clinical trials conducted in human subjects with stroke, leading to U.S. Food and Drug Administration (FDA) approval of paired VNS using the Vivistim® system for upper extremity motor recovery after stroke (35–38). In these studies, vagus nerve afferents were stimulated via direct surgical implantation of electrodes on the left cervical vagus nerve connected to an implantable pulse generator placed in the chest (Vivistim® System, MicroTransponder Inc., Austin, TX).

More recently, Kilgard and colleagues reported a randomized, double-blind, sham-controlled trial evaluating closed-loop vagus nerve stimulation (CLV) paired with intensive upper-extremity rehabilitation in individuals with chronic, incomplete cervical SCI (39). In this study, a novel, miniaturized electrode was implanted on the cervical vagus nerve; however, stimulation was powered and triggered by an external neck-worn module during therapy sessions, rather than by a fully implanted pulse generator. Real-time, movement-contingent stimulation was delivered via therapist- or algorithm-driven triggers, demonstrating promising improvements in upper-extremity strength, function, and activities of daily living. Although the closed-loop approach described by Kilgard et al. enables highly precise, movement-contingent stimulation through automated triggering, it requires specialized external hardware, instrumented rehabilitation equipment, software-based movement sensors, and a neck-worn external power and communication module during therapy sessions (39). This infrastructure may limit scalability and generalizability across rehabilitation settings, as variability in cervical subcutaneous tissue thickness or prior surgical scarring may further affect coupling efficiency and the reliability of stimulation delivery in some individuals.

In contrast, the Vivistim® System employs a fully implantable pulse generator with surgically placed cervical vagus nerve electrodes, allowing consistent, reproducible stimulation delivery without external hardware during therapy. At the time this study was designed, Vivistim® was the only FDA-approved VNS system for pairing stimulation with rehabilitation, supported by randomized controlled trials in stroke populations. Accordingly, our study was designed to evaluate the safety, feasibility, and therapeutic potential of fully implanted invasive VNS (Vivistim®) paired with rehabilitation in individuals with cervical SCI.

Although VNS is already FDA-approved for stroke rehabilitation, evidence in the SCI population remains limited (40). To address this critical unmet need, we conducted a two-arm sham-controlled randomized controlled trial (RCT) to assess recruitment and retention feasibility, evaluate safety, and explore the preliminary effects of pairing active invasive VNS with upper extremity rehabilitation in individuals with cervical SCI.

## Methods

### Study design and participants

This study was a single-site, triple-blind, randomized, sham-controlled pilot trial evaluating the feasibility, safety, and preliminary efficacy of invasive VNS paired with upper extremity rehabilitation in individuals with chronic cervical SCI. The study rationale, design, eligibility criteria, intervention procedures, outcome measures, and statistical analysis plan have been previously published in a peer-reviewed protocol paper and are summarized here (41). Six adults with chronic traumatic incomplete cervical SCI (AIS B–D, neurological level C8 or above) were enrolled and implanted with the Vivistim® System. Participants were randomized in a 1:1 ratio to receive either active VNS paired with rehabilitation or control (sham) VNS paired with rehabilitation. The study design and procedures were modeled on the pivotal VNS trial conducted in the stroke population and were selected to evaluate the feasibility of recruitment, retention, randomization, and blinding for an invasive neuromodulation intervention, rather than to estimate treatment effects (38).

### Recruitment, screening, and baseline assessments

Participants were recruited from TIRR Memorial Hermann outpatient clinics and rehabilitation facilities across the Houston metropolitan area, as well as through community outreach, institutional announcements, and spinal cord injury support and advocacy organizations. Potential participants were screened by the study investigators and research coordinator. All candidates underwent otolaryngology evaluation, including laryngoscopy, to assess vocal cord function prior to enrollment.

Following confirmation of eligibility and informed consent, participants completed pre-implantation and post-implantation baseline assessments. The post-implantation baseline served as the primary reference point for subsequent outcome analyses.

### Device implantation, randomization, and blinding

All participants underwent implantation of the Vivistim® System, consisting of an implantable pulse generator and a cervical vagus nerve electrode. Implantation procedures were performed at Memorial Hermann Hospital by the study neurosurgeon using standard cervical VNS surgical techniques. Participants were observed postoperatively and discharged home the same day. Randomization was performed on the day of implantation in a 1:1 ratio using REDCap. All participants underwent implantation prior to randomization to ensure maintenance of blinding and consistency with prior VNS trials in stroke populations. While this approach exposes all participants to surgical intervention, it was considered necessary to preserve methodological rigor in evaluating paired stimulation paradigms in this early-phase study. Only the research coordinator had access to group assignment and device programming parameters, ensuring blinding of participants, therapists, and outcome assessors.

### Intervention

In-clinic upper-extremity rehabilitation sessions were provided three times per week for 90 min per session over 6-8 weeks (total of 18 sessions), followed by a 90-day home-based rehabilitation program (30-minute sessions on at least 5 days per week). In the active group, VNS was paired with upper extremity task practice during therapy sessions, whereas participants in the control group received sham stimulation during task practice. During in-clinic therapy, stimulation was paired with participants’ active movement attempts, with the therapist triggering stimulation to coincide with task performance. In contrast, during the home-based phase, continuous stimulation during home exercise was delivered in an open-loop manner to support independent use outside the supervised setting. This approach is consistent with prior paired VNS studies in stroke, in which therapist-triggered stimulation during task-specific training in the clinic, followed by continuous stimulation during home exercise, was feasible and associated with improvements in upper-extremity function (35–38). Details about the stimulation parameters, the timing of VNS pairing, and the progression of rehabilitation tasks have been reported previously (41). Participants in the active group received VNS delivered at 0.8 mA, 100 μs pulse width, and 30 Hz frequency. The stimulation pulses lasted 0.5 s at each VNS pairing with movement. The control group received 0 mA pulses. The rehabilitation therapy focused on active movement, task specificity, high-number repetitions, and active participant engagement (41). Tasks were selected from six functional task categories: reach and grasp, gross movement, object flipping, simulated eating tasks, inserting objects, and opening containers. Approximately 30–50 repetitions were performed in each category with a total of 300–500 repetitions per session. To maintain blinding, both groups received identical rehabilitation protocols and initial stimulation procedures at the start of each session.

### Control group cross-over

Following completion of the 90-day follow-up assessment, participants randomized to the control (sham VNS) group were offered the opportunity to cross over and receive six weeks of in-clinic active VNS paired with upper extremity rehabilitation, as specified in the study protocol. Baseline and immediate post-intervention assessments were obtained for participants who elected to cross over. Data from the crossover phase were collected for exploratory purposes and are only included in the safety outcome measure, determined *a priori*.

### Outcome measures

The primary objectives of this pilot study were to assess feasibility and safety. Feasibility outcomes included recruitment, retention, session attendance, and adherence to both in-clinic and home-based rehabilitation. Safety outcomes included surgery and therapy-related adverse events, monitored throughout the study period. Preliminary efficacy was assessed using validated measures of upper extremity motor function, activities of daily living, and quality of life. The primary clinical outcome was the Graded Redefined Assessment of Strength, Sensibility, and Prehension (GRASSP) (42). Secondary outcome measures included the Capabilities of Upper Extremity Questionnaire (CUE-Q) (43), as detailed in the protocol paper (41). For the GRASSP, prior work suggests that changes of approximately 4–6 points may represent minimal clinically important differences (MCIDs) in individuals with cervical SCI; therefore, a ≥ 6-point change was used as a conservative threshold for exploratory responder analyses (39, 44). For the CUE-Q, an MCID has not been established, and changes are interpreted descriptively. Additionally, the participant satisfaction and treatment tolerability were assessed using a structured Patient Satisfaction Questionnaire completed by each participant at the end of the intervention period. The questionnaire evaluated overall satisfaction with the assigned study treatment, perceived change in overall health and well-being, and satisfaction with tolerability of treatment-related side effects using 5-point ordinal Likert-type response options (ranging from very satisfied to very dissatisfied). This measure was included to assess treatment acceptability and perceived benefit in this pilot study.

### Statistical analysis

Given the pilot nature of the study, analyses were primarily descriptive. Outcomes were summarized by treatment group following the intention-to-treat principle. Continuous variables were summarized using means and standard deviations or medians and interquartile ranges, as appropriate, and categorical variables were summarized using frequencies and percentages. Given the small sample size, analyses were not intended for formal inference, and estimation of variability, confidence intervals, or effect sizes was not emphasized, as such estimates would be unstable and potentially misleading. A detailed statistical analysis plan is provided in the published protocol.

## Results

### Participant enrollment and demographics

**Figure 1:**
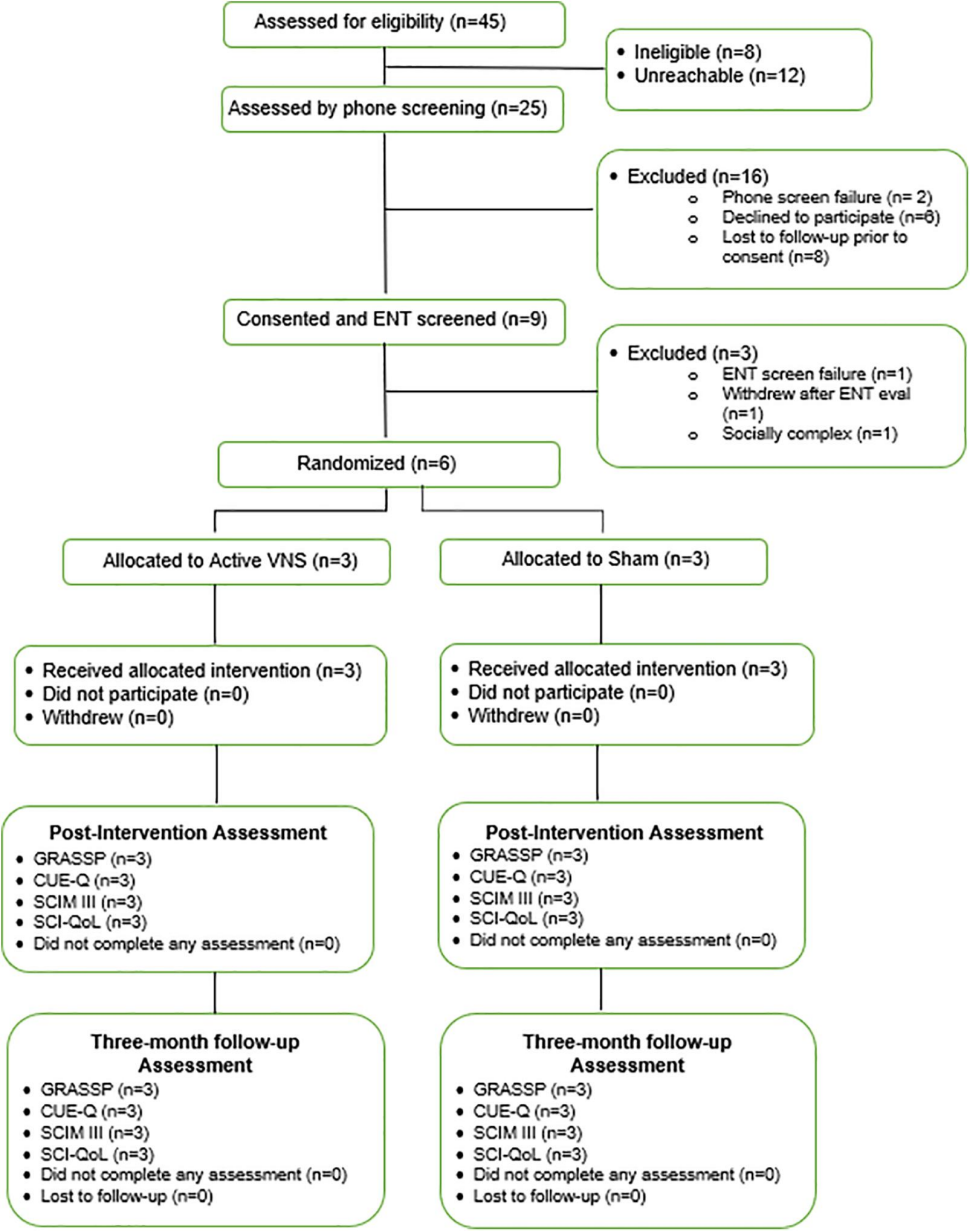
CONSORT flow diagram of participant screening, enrollment, allocation, follow-up, and analysis. The diagram illustrates participant flow through eligibility assessment, randomization to active or sham paired vagus nerve stimulation (VNS), intervention completion, and post-intervention and three-month follow-up assessments.

Six participants with chronic traumatic incomplete cervical SCI were implanted with the Vivistim® System. Enrollment was closed after six participants due to budgetary constraints, even though the investigational device exemption (IDE) approval allowed up to eight implants. All implanted participants completed the study procedures through the 90-day follow-up. No participants withdrew from the study after implantation. All participants in the control (sham) group elected to cross over to active stimulation after completing the control phase, at varying time points following study completion. Participant demographic and injury characteristics are summarized in Table 1. The study included six adults with chronic traumatic incomplete cervical SCI (AIS B–D) with cervical neurological levels ranging from C3 to C5 and a wide range of time since injury (Figure 1).

**Table 1:** Demographic and injury characteristics of study participants.

Participant ID	Age (yrs)	Sex	Ethnicity	Neurological level of injury	AIS Grade	Time since injury (mo)	Cause of injury
001	67	Male	Hispanic or Latino	C4	C	49	MVA
002	27	Male	Hispanic or Latino	C5	B	54	MVA
003	51	Male	Not Hispanic or Latino	C3	C	20	Fall into a swimming pool
004	44	Female	Not Hispanic or Latino	C3	D	61	MVA
005	39	Male	Not Hispanic or Latino	C5	C	43	MVA
006	41	Male	Not Hispanic or Latino	C4	D	16	Dive into a shallow swimming pool

AIS, American Spinal Injury Association impairment scale; MVA, motor vehicle accident.

### Safety outcomes

#### Surgical and device safety

No serious adverse events, deaths, unanticipated adverse device effects, or unresolved device- or surgery-related complications were observed during the study period (Table 2).

**Table 2:** Safety data: adverse events.

Adverse event type	Active (*n* = 3)	Control (*n* = 3)	Control cross- over (*n* = 3)
VNS surgery-related unresolved at 90-day f/u	0	0	-
VNS Device-related unresolved complication at 90-day f/u	0	0	-
VNS therapy-related	Number of episodes		
Cough during stimulation	3	1	1
Bladder spasms/ discomfort	0	1	1

One participant experienced mild neck pain and erythema at the surgical site, which resolved within one week without intervention. No cases of vocal cord paralysis, dysphagia, infection, or other surgery-related complications were reported through the 90-day follow-up. Device-related events were rare. One participant reported subjective sensations of stimulation outside therapy sessions; device interrogation confirmed no unintended stimulation events. The participant declined device deactivation or removal, and symptoms resolved spontaneously without further intervention. No unresolved device-related adverse events occurred.

#### Paired VNS therapy safety

VNS therapy-related adverse events were mild and transient (Table 2). Four participants reported a brief cough during stimulation in 6 of 97 total in-clinic therapy sessions (0.06%). Cough occurred only during early sessions, resolved spontaneously, and did not recur. One participant in the control group reported bladder spasms during therapy; these were attributed to ongoing urologic issues unrelated to stimulation and resolved following suprapubic catheter exchange. No episodes of autonomic dysreflexia, persistent pain, or worsening spasticity attributable to VNS were observed.

### Feasibility outcomes

#### In-clinic therapy adherence

Feasibility benchmarks for in-clinic rehabilitation were met (Table 3, Figure 1). Four of six participants completed all 18 in-clinic rehabilitation sessions. One participant missed a single session, and one participant completed 8 of 18 sessions due to transportation issues related to a caregiver's health; however, this participant remained engaged in the home-based rehabilitation program. Overall adherence exceeded the predefined threshold of 80%, with five participants completing ≥94% of scheduled sessions (Table 3).

**Table 3:** Feasibility outcomes for in-clinic therapy sessions and post-intervention home exercise adherence.

Participant ID	Group	In-clinic therapy sessions, *n* (%)	Average paired stimulations per in-clinic session	Post-intervention 90-day home exercise program, *n* (%)
001	Active	18 (100%)	419	61 (95%)
002	Sham	18 (100%)	430	16 (25%)
003	Active	18 (100%)	405	49 (77%)
004	Sham	8 (44%)	353	27 (42%)
005	Sham	17 (94%)	354	30 (47%)
006	Active	18 (100%)	427	42 (64%)
Overall		16 (90%)	398	38 (59%)

n, number of sessions completed or days adhered, as applicable. Percentages reflect proportion of days completed.

Participants in the active and sham groups received a comparable number of paired stimulations per in-clinic rehabilitation session. The mean number of vagus nerve stimulations per session was 417 in the active VNS group and 379 in the sham group, for an overall mean of 398.

#### Home exercise program adherence

Adherence to the 90-day home exercise program varied across participants, ranging from 25% to 95% of expected sessions, with an average of 38 sessions (59% of recommended sessions) over 90 days (Table 3). Despite this variability, all participants engaged in home-based rehabilitation to some degree, and no attrition occurred during the follow-up period.

### Clinical outcomes

**Figure 2:**
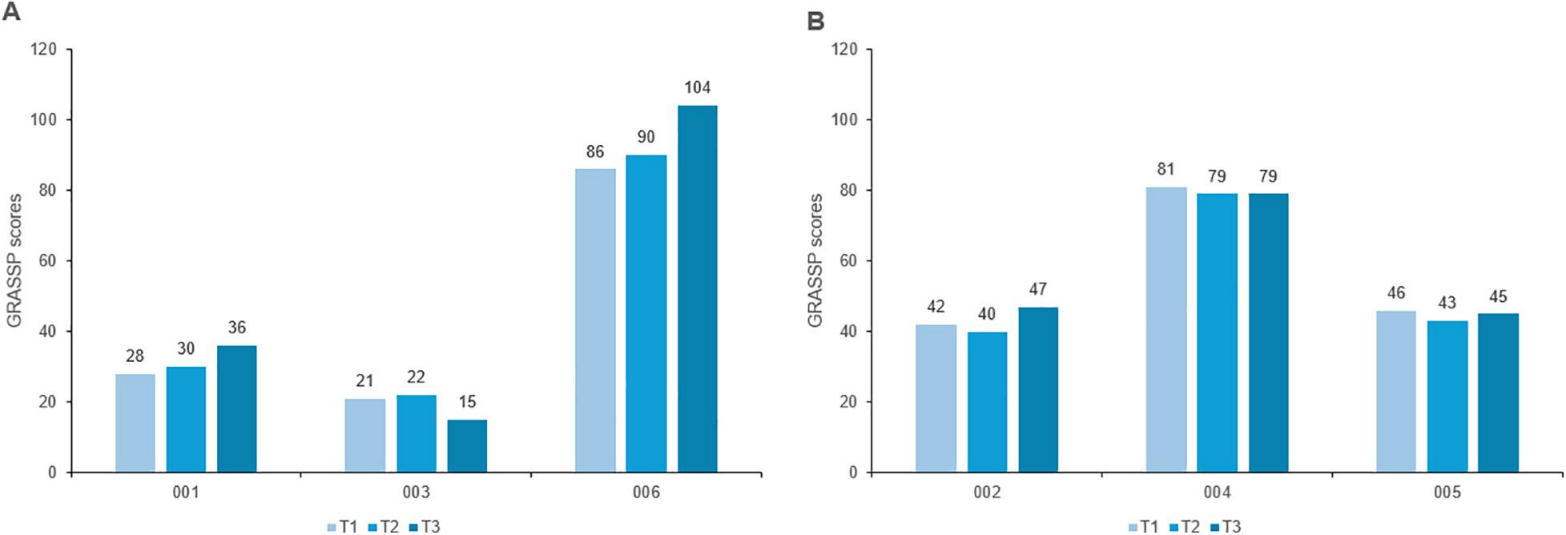
GRASSP total scores of the trained upper extremity across study time points. Upper extremity function measured using Graded Redefined Assessment of Strength, Sensibility and Prehension (GRASSP) scores for **(A)** participants receiving active paired vagus nerve stimulation (VNS; participants 001, 003, and 006) and **(B)** participants receiving sham paired VNS (Participants 002, 004, and 005). Individual scores are shown at baseline (T1), post-intervention (T2), and 90-day follow-up (T3). Bars represent individual participant values.

This pilot study was primarily designed to evaluate the safety and feasibility of pairing VNS with upper extremity rehabilitation in individuals with chronic cervical SCI, with clinical outcomes assessed as exploratory endpoints. At the immediate post-intervention assessment following completion of in-clinic therapy, all three participants randomized to the active VNS group demonstrated improvement in total GRASSP scores compared with baseline (Figure 2). In contrast, none of the three participants in the control (sham VNS) group demonstrated improvement in total GRASSP scores at the post-intervention assessment.

**Figure 3:**
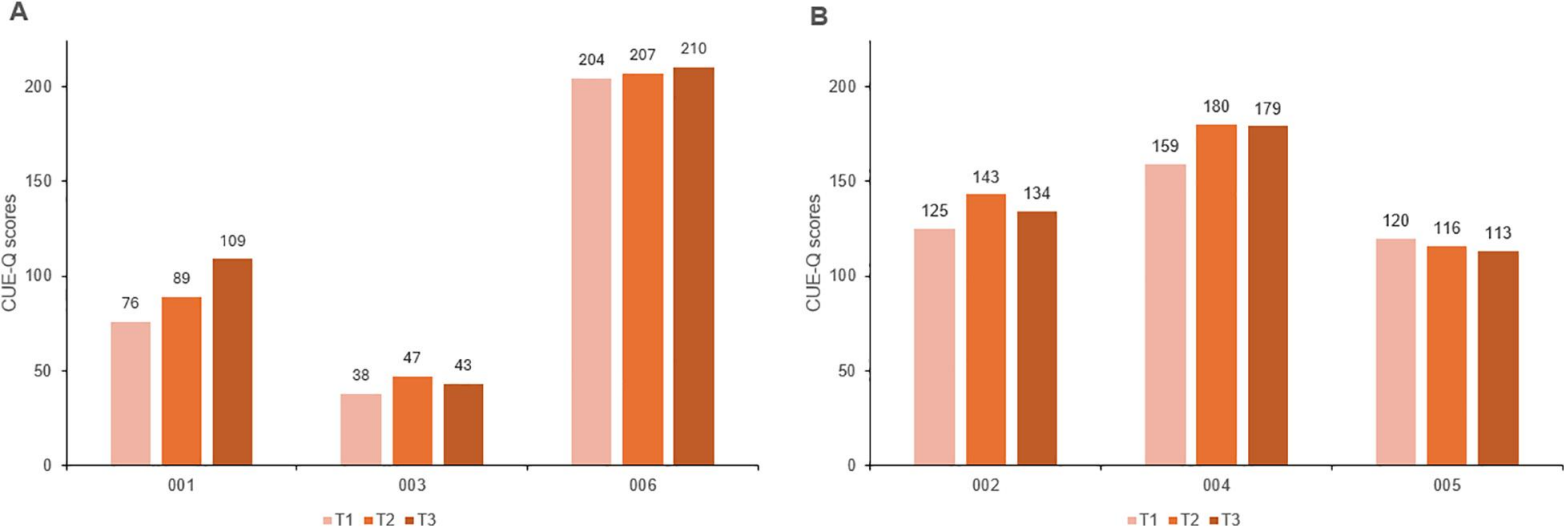
Capabilities of upper extremity questionnaire (CUE-Q) scores across study time points. Patient-reported Capabilities of Upper Extremity Questionnaire (CUE-Q) scores are shown at baseline (T1), post-intervention (T2), and 90-day follow-up (T3) for **(A)** participants receiving active paired vagus nerve stimulation (VNS) and **(B)** participants receiving sham paired VNS. Bars represent individual participant values.

At the 90-day follow-up, two of the three participants in the active VNS group sustained improvements in GRASSP scores relative to baseline, whereas one participant showed attenuation of the initial gains. In the control group, one participant demonstrated improvement in the GRASSP score by the 90-day follow-up, while the remaining two participants showed no improvement (Figure 2). When interpreted using a clinically meaningful threshold (≥ 6-point improvement), two of three participants in the active group met this threshold at follow-up, whereas none of the participants in the control group achieved this level of improvement. Given the small sample size, these findings are descriptive and should be interpreted cautiously. Changes in secondary functional (Figure 3) and patient-reported outcomes were heterogeneous and are interpreted descriptively, given the small sample size of this pilot study.

In addition to standardized outcome measures, participants in the active VNS group reported subjective improvements in upper extremity function following the intervention. Reported changes included improved coordination and movement smoothness, increased pinch strength, and greater ease in performing activities of daily living such as eating with utensils, drinking from a cup using one upper extremity, signing a check, and carrying lightweight objects independently. Although these qualitative observations were not consistently captured by standardized outcome measures, they reflect functional changes that participants perceived as meaningful in daily life and provide complementary context to the quantitative findings of this pilot study (Supplementary Table S1).

### Participant satisfaction survey

All participants (*n* = 6) reported positive satisfaction with their assigned study treatment and protocol. Overall treatment satisfaction was high, with 4 participants reporting being *very satisfied* and 2 reporting being *satisfied*; no neutral or negative responses were reported. Perceived changes in overall health and well-being were uniformly favorable, with half of the participants (*n* = 3) reporting being *much improved* and the remaining half (*n* = 3) reporting being *improved* since initiating the study treatment. Tolerability of treatment-related side effects was also rated highly: 5 participants reported being *very satisfied,* and one reported being *satisfied*. No participants reported difficulty tolerating side effects, and no adverse effects were noted in the sham group. Satisfaction and perceived benefit were comparable between the active and sham groups.

## Discussion

This triple-blind, randomized, sham-controlled pilot study evaluated the safety and feasibility of pairing invasive VNS with upper extremity rehabilitation in individuals with chronic cervical SCI. The primary objectives were to establish procedural safety and feasibility; exploratory clinical outcomes were included to inform the design of future trials rather than to test efficacy. Hence, all clinical outcomes should be interpreted as exploratory and hypothesis-generating. Overall, the study demonstrates that implantation of the FDA-approved Vivistim® VNS system and its integration with intensive rehabilitation is safe, well-tolerated, and feasible in the SCI population.

No serious adverse events, unanticipated adverse device effects, or unresolved surgery-related complications were observed through 90 days of follow-up. Both the implantation procedure and subsequent in-clinic and home-based rehabilitation phases were tolerated without clinically significant autonomic, respiratory, or laryngeal complications. Therapy-related adverse events were infrequent, mild, and transient. These findings extend the established safety profile of invasive VNS previously demonstrated in epilepsy and stroke to individuals with chronic cervical SCI, a population with unique physiological vulnerabilities.

Feasibility benchmarks were met for in-clinic rehabilitation, with high adherence to scheduled therapy sessions and no post-implantation attrition. The stimulation dose and session duration in this study were comparable to those reported in pivotal VNS stroke rehabilitation trials, supporting protocol fidelity and translational relevance. However, transportation and access barriers emerged as meaningful challenges, affecting recruitment and, in one case, limiting completion of in-clinic sessions (only 8 sessions) despite continued engagement in home-based therapy. These observations highlight the real-world constraints faced by individuals with SCI and underscore the need for more accessible, flexible rehabilitation delivery models.

Although this pilot study was not powered to assess efficacy, exploratory clinical outcomes suggested a pattern of improvement in the active VNS group. All participants receiving active VNS demonstrated mild-to-moderate improvements in GRASSP scores immediately after completing in-clinic therapy, whereas no participants in the sham group showed post-intervention improvement. At the 90-day follow-up, two of the three participants in the active group achieved changes consistent with clinically meaningful improvement in GRASSP scores compared to none in controls. However, these findings should be interpreted cautiously given the small sample size and exploratory nature of the analysis. The primary contribution of this study remains the demonstration of safety, tolerability, and feasibility. In the control group, clinical changes over time were variable and inconsistent. The partial attenuation of post-intervention gains in some participants may reflect several factors. Transition from supervised, high-intensity in-clinic therapy to a largely self-directed home exercise program may reduce consistency, intensity, or task specificity of training. Adherence to the home exercise program was variable across participants, which may have influenced the durability of functional gains. While stimulation delivery and rehabilitation intensity were prescribed and implemented according to protocol across groups, granular analyses of cumulative stimulation exposure and therapy dose are beyond the scope of this pilot report. These observations suggest that sustained benefits may require longer in-clinic therapy periods, enhanced support during the home phase, or hybrid models incorporating tele-rehabilitation and more frequent therapist engagement. The exploratory signal observed in this study is biologically plausible and aligns with prior mechanistic and clinical literature demonstrating that precisely timed VNS paired with motor practice can enhance activity-dependent neuroplasticity (35–40, 45). In a recent double-blind RCT (*n* = 19) by Kilgard et al., participants receiving CLV achieved a statistically significant 4.1 ± 1.5-point improvement in total GRASSP scores over 36 sessions. Furthermore, 36 sessions produced accumulating gains nearly double those observed after 18 sessions, suggesting a dose-dependent response. The dose of rehabilitation and cumulative stimulation exposure appear to be critical determinants of the magnitude of gains and their durability. Differences in stimulation delivery precision may further modulate effective dose. Kilgard et al. employed a closed-loop system in which stimulation was triggered in real time based on movement kinematics, ensuring close temporal coupling between VNS delivery and motor attempts. While this closed-loop approach offers advantages in precision, it requires specialized external hardware and sensorized rehabilitation tools that may limit scalability across diverse clinical settings. In contrast, the present study evaluated a fully implanted VNS system that enables consistent stimulation delivery without reliance on external hardware during therapy. This approach may offer advantages in reliability, clinical workflow integration, and broader applicability, particularly in individuals with variable cervical anatomy, subcutaneous tissue thickness, or prior surgical scarring. This clinician-triggered paired stimulation approach, followed by continuous stimulation during home exercise, was adapted from prior stroke pilot and pivotal studies of paired VNS, in which this approach was shown to be feasible and associated with functional improvements (35–38). Accordingly, the present study should be viewed as complementary to closed-loop approaches, focusing on evaluating a fully implantable, clinically scalable system that can be more readily integrated into routine rehabilitation settings. Collectively, these complementary studies support the feasibility of VNS-enhanced rehabilitation in SCI while highlighting the need to define optimal stimulation paradigms, optimal dose-response relationship, patient selection criteria, and implementation strategies. Future studies should explore automated or technology-assisted stimulation approaches to improve scalability and precision.

Participant-reported experiences further emphasize the importance of outcome measures that capture meaningful functional change. Several participants described improvements in coordination, smoothness of movement, pinch strength, and independence in everyday tasks such as signing a check, eating with utensils, drinking from a cup using one upper extremity, and carrying shopping bags that were not consistently reflected in standardized assessments (Supplementary Table S1). Incorporating quantitative movement analyses and patient-centered outcome measures may provide a more comprehensive understanding of recovery in future trials.

### Limitations

This study has several important limitations. First, the small sample size and pilot design limit generalizability, preclude formal statistical comparisons, and restrict the ability to reliably estimate variability, confidence intervals, or effect sizes, reinforcing that the findings should be interpreted descriptively rather than inferentially. Clinical outcomes were exploratory and should not be interpreted as evidence of efficacy. Second, participants demonstrated a wide range of baseline upper-extremity function, from very low to relatively high. The inclusion of participants across AIS B–D and varying chronicity introduces clinical heterogeneity that limits interpretation of between-group differences and further reinforces the exploratory nature of outcome findings. Future studies should consider refining eligibility criteria or employing stratified randomization based on baseline function.

Third, transportation and access barriers affected recruitment and participation, highlighting a need for more flexible and accessible rehabilitation models. Fourth, adherence to the home exercise program was variable, and although stimulation delivery and rehabilitation intensity were protocolized, exercise performance during home sessions was not directly monitored. Fifth, management of spasticity with botulinum toxin or phenol injections was necessary in some participants to support participation, reflecting real-world clinical practice but potentially influencing outcomes. Future trials will need clearly defined policies for spasticity management to balance patient care with methodological rigor. Finally, while control participants were offered crossover to active VNS after the 90-day follow-up, crossover data were collected for exploratory and ethical purposes only and were not included in primary analyses.

## Conclusion

In this pilot randomized controlled trial, pairing invasive vagus nerve stimulation with upper extremity rehabilitation in individuals with chronic cervical spinal cord injury was safe, feasible, and well tolerated. High adherence to in-clinic therapy and the absence of serious or unanticipated adverse events support the practicality of this intervention in a clinical rehabilitation setting. Although clinical improvements were mild to moderate and observed in a small number of participants, consistent post-intervention gains in the active VNS group suggest a potential signal worthy of further investigation. These findings extend the safety profile of invasive VNS to the SCI population and provide foundational human data to inform future studies. Larger, adequately powered trials incorporating refined inclusion criteria, enhanced support for home-based therapy, longer or more intensive in-clinic rehabilitation, quantitative movement assessments, and hybrid in-clinic/tele-rehabilitation models will be essential to determine efficacy, durability, and optimal implementation of VNS paired with rehabilitation in spinal cord injury.

## Data Availability

The datasets generated and analysed during the current study are available from the corresponding authors upon reasonable request, in accordance with UT Health Houston data sharing policies and applicable regulatory requirements.
