# Supplementary material for: Safety and feasibility of paired vagus nerve stimulation with rehabilitation for improving upper extremity function in people with cervical spinal cord injury: a pilot randomized controlled trial
Source: Front Rehabil Sci. 2026 Jun 11;7:1805955. doi: 10.3389/fresc.2026.1805955 (PMC13294046; doi:10.3389/fresc.2026.1805955)
Supplement: Supplementary file 1 [file Table1.docx]

**Supplementary Table S1. Qualitative participant-reported observations during the study intervention and follow-up**

| **Participant ID** | **During the in-clinic study intervention** | **At follow-up** |
| --- | --- | --- |
| 001 | No qualitative observations reported. | Reported ability to write with the left hand, which had not been previously possible. Caregiver noted overall functional improvements (e.g., folding towels). |
| 002 | Reported subjective improvements in signing checks and removing cards from a wallet.  Reported feeling stronger in right hand. | Reported faster and more natural ability to insert a key into a lock; reported improved handwriting with reduced hand fatigue. |
| 003 | No qualitative observations reported. | Reported ability to pick up food with fork during inpatient phase 2 (4 weeks total). Reported improved knee movement and increased sensation following in-clinic sessions, noted temperature sensation in knee and thigh during showering. |
| 004 | No qualitative observations reported. | Reported increased ability to perform small household tasks. |
| 005 | Reported feeling increased "push" strength in right upper extremity. | Reported ability to carry out tasks requiring more strain that were not previously possible; reported noticeable strength improvements. |
| 006 | Reported increased coordination in bilateral upper extremities, including pad-to-pad pinch with left hand.  Noted increased fine motor control bilaterally, specifically left thumb strength, grip strength, and improved mobility in neck and shoulders.  Reported new temperature sensation in left foot. | Reported feeling new temperature sensation in left leg and strength improvement in left thumb. Reported most rapid improvement in the first six weeks, including new functional abilities not previously possible (e.g., holding utensils and clipping toenails), with continuing improvement in tasks thereafter. |

**Abbreviations:** None.
